# Precision medicine in psychosis: Translating findings from research into clinical practice

**DOI:** 10.1192/j.eurpsy.2021.218

**Published:** 2021-08-13

**Authors:** S. Galderisi

**Affiliations:** Department Of Psychiatry, University of Campania “Luigi Vanvitelli”, NAPOLI, Italy

## Abstract

**Abstract Body:**

Precision medicine is “an emerging approach for treatment and prevention that takes into account each person’s variability in genes, environment, and lifestyle” [1]. The terminology is increasingly used in psychiatry, and especially in research relevant to the prediction of psychosis onset, response to treatment and functional outcome. While this is an important step-forward for the discipline, at this stage it is very important to promote the translation of research findings into clinical practice, as much as possible. Nowadays the availability of machine learning and artificial intelligence tools, together with advances in data storage and data security, enable the integration of neuroimaging, biological, clinical and cognitive data. By overcoming current limitations in multiple domain data analysis these tools may lead to the identification of reliable diagnostic, prognostic and therapeutic markers in routine clinical care, as well as to the prediction of clinically meaningful outcomes (e.g., psychosis onset, symptomatic and functional outcome, and treatment response). Precision medicine in psychiatry is a developing science, deserving further large-scale research, translational approaches and refinement that, hopefully, will soon be an integral part of every-day clinical practice. However, challenges in pursuing this strategy should not be underestimated, and efforts should be made to constantly advocate for more investments in human and financial resources in psychiatry, and to concentrate on the use of widely available and not too expensive and time-consuming methods.^1^ Toward Precision Medicine. Building a Knowledge Network for Biomedical Research and a New Taxonomy of Disease. Washington, DC: National Academies Press; 2011.

**Disclosure:**

No significant relationships.

